# Potential predictive markers of chemotherapy resistance in stage III ovarian serous carcinomas

**DOI:** 10.1186/1471-2407-9-368

**Published:** 2009-10-18

**Authors:** Lovisa Österberg, Kristina Levan, Karolina Partheen, Ulla Delle, Björn Olsson, Karin Sundfeldt, György Horvath

**Affiliations:** 1 Department of Oncology, Institute of Clinical Sciences, University of Gothenburg, Sweden; 2 School of Life Sciences, University College of Skövde, Sweden; 3 Department of Obstetrics and Gynecology, Institute of Clinical Sciences, University of Gothenburg, Sweden

## Abstract

**Background:**

Chemotherapy resistance remains a major obstacle in the treatment of women with ovarian cancer. Establishing predictive markers of chemoresponse would help to individualize therapy and improve survival of ovarian cancer patients. Chemotherapy resistance in ovarian cancer has been studied thoroughly and several non-overlapping single genes, gene profiles and copy number alterations have been suggested as potential markers. The objective of this study was to explore genetic alterations behind chemotherapy resistance in ovarian cancer with the ultimate aim to find potential predictive markers.

**Methods:**

To create the best opportunities for identifying genetic alterations of importance for resistance, we selected a homogenous tumor material concerning histology, stage and chemotherapy. Using high-resolution whole genome array comparative genomic hybridization (CGH), we analyzed the tumor genomes of 40 fresh-frozen stage III ovarian serous carcinomas, all uniformly treated with combination therapy paclitaxel/carboplatin. Fisher's exact test was used to identify significant differences. Subsequently, we examined four genes in the significant regions (*EVI1*, *MDS1*, *SH3GL2*, *SH3KBP1*) plus the *ABCB1 *gene with quantitative real-time polymerase chain reaction (QPCR) to evaluate the impact of DNA alterations on the transcriptional level.

**Results:**

We identified gain in 3q26.2, and losses in 6q11.2-12, 9p22.3, 9p22.2-22.1, 9p22.1-21.3, Xp22.2-22.12, Xp22.11-11.3, and Xp11.23-11.1 to be significantly associated with chemotherapy resistance. In the gene expression analysis, *EVI1 *expression differed between samples with gain versus without gain, exhibiting higher expression in the gain group.

**Conclusion:**

In conclusion, we detected specific genetic alterations associated with resistance, of which some might be potential predictive markers of chemotherapy resistance in advanced ovarian serous carcinomas. Thus, further studies are required to validate these findings in an independent ovarian tumor series.

## Background

In advanced epithelial ovarian cancer, current standard first-line chemotherapy is platinum- and taxane-based; most frequently in the form of carboplatin and paclitaxel. Most patients initially respond to this chemotherapy (60-80%), but the majority eventually recurs with chemoresistant tumor and succumbs to metastatic disease [1,2]. Thus, ovarian cancer is the most lethal gynecologic malignancy with a five-year survival of around 30% in advanced stage disease; about 70-80% of patients are diagnosed with advanced stages [3]. Finding predictive markers of chemoresistance and elucidating resistance mechanisms is hence crucial for individualizing and improving treatment and survival of ovarian cancer patients. Drug resistance in ovarian cancer is extensively studied and has proved to be complex, occurring at different cellular levels as well as on a pharmacological level. The frequently used chemotherapy paclitaxel exerts its cytotoxic effect by binding to β-tubulin, thereby stabilizing the microtubules and inducing apoptosis [4]. Multiple resistance mechanisms have been suggested for paclitaxel; such as alterations of tubulin/microtubules, altered signaling pathways of the cell cycle and apoptosis, and over expression of multidrug efflux pumps [5,6]. The platinum agent carboplatin induces apoptosis by forming platinum-DNA adducts [7]. Carboplatin resistance mechanisms include decreased net intracellular drug accumulation, drug detoxification, enhanced DNA repair mechanisms, or changes in apoptotic signaling pathways [8-11].

Genetic changes such as copy number alterations (CNAs) are important in tumor development, and therefore most likely of importance for chemotherapy resistance as well. A useful key technique to study CNAs with is the array format of comparative genomic hybridization (CGH), a high-resolution genome-wide screening method that detects and maps copy number changes in the tumor genome. There are a few reports utilizing array CGH when studying chemotherapy resistance in ovarian cancer [12-15], and in addition there are a number of reports performed with conventional metaphase CGH [16-19]. Unfortunately, the overall concurrence is low, pin-pointing the need of further studies.

Even though taxane- and platinum resistance has been greatly studied there is still much to elucidate. In the present investigation, we sought to identify genetic alterations of importance for chemotherapy resistance in advanced ovarian cancer, with the ultimate aim to uncover predictive markers. We selected a homogenous primary tumor material concerning histology, stage and chemotherapy response to create the best opportunities for identifying genetic alterations of importance for resistance. High-resolution whole genome array CGH was used to scan tumor genomes of fresh-frozen stage III ovarian serous carcinomas. Subsequently, we examined five genes (*EVI1*, *MDS1*, *SH3GL2*, *SH3KBP1*, and *ABCB1*) with quantitative real-time polymerase chain reaction (QPCR) to explore the impact of DNA alterations on the transcriptional level.

## Methods

### Tumor material

Forty stage III epithelial ovarian serous papillary carcinomas were analyzed with array CGH (Table 1; Additional file 1:Clinical characteristics). The tumors were collected at the time for primary debulking surgery and stored in -80°C until analysis. All patients were, following surgery, uniformly treated with combination chemotherapy paclitaxel/carboplatin. Patients were defined as clinically resistant when they had steady disease or progressive disease after first-line chemotherapy, or recurrent disease within six months after the last administration of first-line chemotherapy. Twenty patients were resistant according to these criteria. Patients were defined as clinically sensitive when they had clinical complete remission after first-line chemotherapy (20 patients). All 20 sensitive cases survived more than five years from diagnosis, and all resistant cases were primary resistant.

**Table 1:** Clinicopathologic characteristics

	No.	r/s
**Chemotherapy response**		
Resistant	20	
Sensitive	20	

**Survival**		
Deceased	20	
Survivors	20	

**Histology**		
Serous	40	

**FIGO stage**		
Stage III	40	

**FIGO grade**		
Well	8	2/6
Moderately	11	8/3
Poorly	21	10/11

**Tumors used in QPCR**	17	8/9

**Total**	40	

Clinicopahtologic characteristics of the ovarian tumor material from the 40 patients. Distribution of resistant (r) and sensitive (s) cases respectively is shown in the third column.

The tumors were classified histologically using standard World Health Organization (WHO) criteria, and clinical staging and tumor grading was performed according to the International Federation of Gynecology and Obstetrics (FIGO) standards. All tumors were assessed by one pathologist, according to regional treatment guidelines for gynecological malignancies in western Sweden. In addition, specimen imprints for cytologic evaluation were performed to verify the presence of tumor cells (stained with May-Grünwald-Giemsa stain). At least 70% tumor cells content was required for each tumor specimen. The tumors investigated were collected from patients diagnosed between 1995 and 2003 at Sahlgrenska University Hospital in Gothenburg, and the study was approved by the local ethics committee. Median age of the patients at initial diagnosis was 59.5 years (range 40-79 years), and median follow-up time was 7 years (range 5-11 years) among survivors.

### Array CGH

Tiling, whole genome coverage BAC arrays (38,043 BAC clones) were produced at the SCIBLU Genomics Center, Department of Oncology, Lund University, Sweden http://www.lth.se/sciblu as previously described [20]. BAC clones were mapped to the hg17 genome build. Array CGH was performed essentially as previously described [20]. Normal female reference DNA containing a mix from ten healthy individuals was purchased from Promega, Madison, WI, USA. Identification of individual spots on scanned arrays was performed with GenePix Pro software 6.0.1.12 (Axon Instruments, Union City, CA, USA), and the quantified data matrix was loaded into the web-based database Bio Array Software Environment (BASE) http://base.thep.lu.se/[21].

#### Data analysis

Background correction of Cy3 and Cy5 intensities was calculated using median-feature and median-local background, generating test over reference log_2 _ratios. Flagged features were removed and spots that had background-corrected Cy3 or Cy5 intensities < 0 or > 65000 were removed from further analysis. A signal-to-noise filter of ≥ 5 for both tumor and reference channels was applied to the data. The filtered data were normalized using the popLowess algorithm [22]. After normalization, a smooth was applied with a moving median sliding window of 250 kbp, and with adaptive thresholds [22]. The CGH-Plotter software [23], as an R http://www.r-project.org implementation in BASE, was used for segmentation. The segmentation constant, c, was set to 8. Copy number alterations were determined by comparing the segmented log_2 _ratios to gain/loss thresholds obtained by an adaptive scaling method [22], using a window size of 2% and a scaling factor of 2. Segments were accordingly designated gained, lost or not changed, giving a ternary scale (-1, 0, 1). Using the values given by CGH-plotter the frequency of copy number changes per tumor was calculated (alterations defined as >3 adjacent clones). To facilitate any cross-platform comparison, segmented data was transformed into a virtual probe set with probes spaced at every 50 kbp throughout the entire genome by associating each probe to its closest virtual probe [24].

#### Statistical analysis

Two-tailed Fisher's exact test was used to identify gains or losses that differed significantly in frequency between the sensitive and resistant tumor groups. A cutoff value of *P *< 0.01 was used to reduce the effect of multiple testing in this large data set. Further, it has been demonstrated that analyzing segmented data greatly increases the power to detect true significant associations without increasing the false discovery rate [25]. Gains were tested against no gain and losses were tested against no loss. To test if the frequency of altered genome differed between the groups, one-tailed Student's t-test was performed. A decision tree for classifying samples as resistant or sensitive was generated based on the significant regions using the J48 algorithm in the Weka software. Tests included in the resulting tree were based on binary decisions, i.e. gain versus no gain and loss versus no loss. The algorithm was applied with the default settings of Weka version 3.6.0 [26]. Classification accuracy on samples was estimated using n-fold leave-one-out cross validation. The decision tree was further tested on another published data set of 98 stage III ovarian serous tumors with survival as endpoint (29 survivors versus 69 non-survivors) [27], to evaluate our findings. Non-survivors were regarded as equivalent to resistant and survivors to sensitive in the tree. Survival curves of the significant regions were prepared using the Kaplan-Meier method in the SPSS software, version 16 (Superior Performance Software System, SPSS for Windows, Chicago, IL, USA) and *P*-values for the difference between the curves were calculated using the Breslow-Wilcoxon test [28].

### Quantitative real-time polymerase chain reaction (QPCR)

QPCR was performed on 17 cases, eight resistant and nine sensitive, to explore gene expression of the five genes *EVI1*, *MDS1*, *SH3GL2*, *SH3KBP1*, and *ABCB1*. QPCR was essentially performed as previously described [29]. In brief, total RNA was isolated from all 40 fresh-frozen ovarian tumors by homogenization with TRIzol Reagent (Invitrogen, Carlsbad, CA, USA), and then extracted with RNeasy mini kit (Qiagen, Valencia, CA, USA). After quality control of RNA using the 2100 bioanalyzer (Agilent Biotechnologies, Palo Alto, CA, USA), 17 samples passed and were further analyzed (RNA integrity number value: median 6.6, range 4.7-7.9). From each tumor sample, 1 μg total RNA was reverse transcribed in duplicate, as well as negative controls without enzyme, using the iScript kit (Bio-Rad Laboratories, Hercules, CA, USA). Each cDNA sample was analyzed in triplicate by real-time PCR, and detected with PerfeCTa SYBR Green Supermix (Quanta Biosciences, Gaithersburg, MD, USA). Two reference genes were used, *GAPDH *and β-actin, both previously found to be stably expressed in ovarian tumor material [29]. Primer sequences for the target genes are presented in Additional file 2:Primer sequences. Reference gene assays used for normalization were obtained from the Human Endogenous Control Gene Panel (TATAA Biocenter, http://tataa.com). The efficiency of each QPCR assay was estimated from the slope of a standard curve generated from the serial dilution of purified PCR products. When analyzing the QPCR data, samples were grouped according to the corresponding CNA exhibiting significance in the array CGH analysis (*EVI1*, *MDS1*, *SH3GL2*, *SH3KBP1*) or according to chemotherapy response (*ABCB1*), and a one-tailed Student's t-test was performed between the groups.

## Results

### Array CGH

Using high-resolution whole genome array CGH, we explored copy number alterations in 40 stage III ovarian serous carcinomas in relation to chemotherapy response. The amount of alterations detected by array CGH varied greatly between the samples. On average, the frequency of altered genome was 32% per tumor, and the size of altered genome per tumor was 946 Mbp (range 42-2137 Mbp). The majority of the samples exhibited a simplex genome pattern, i.e. rather large low-level alterations; however, smaller changes were also abundant. Overall, low-level copy number alterations dominated, homozygous deletions were scarce, and high-level amplifications were not very frequent. The most frequent CNAs in the total material were gains in 1q, 3q, 8q, 20pq, and losses in 4q, 8p, 17p and Xpq. The original array CGH data are available in Additional file 3:Array data.

**Figure 1:**
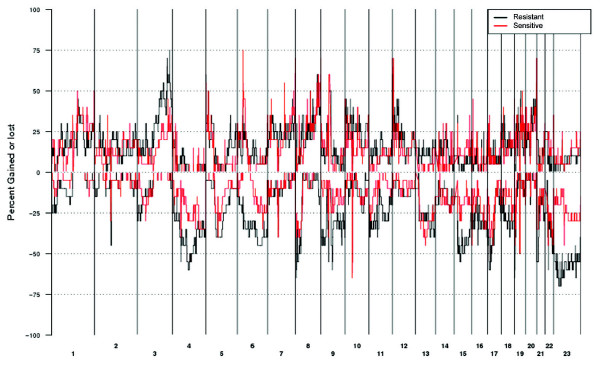
**Genome-wide frequency plot**. Genome-wide frequency plot of the CNAs in the total ovarian tumor material consisting of 40 tumors. Genomic fragments are in chromosomal order as indicated; gains are upwards and losses downwards. Black line represents resistant cases, and red line sensitive cases.

**Figure 2:**
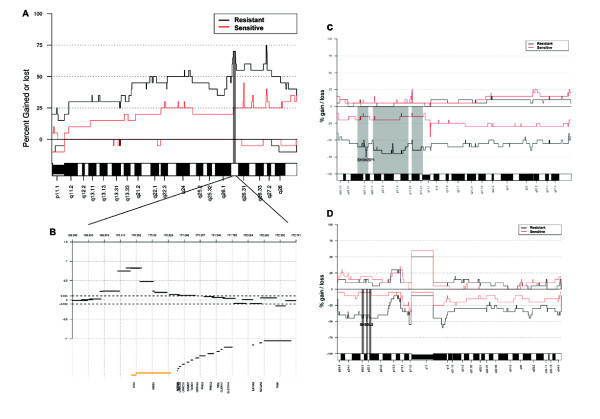
**Frequency plots**. A) Frequency plot of chromosome arm 3q. The significant region in 3q26.2 is highlighted in grey. B) Example of one case exhibiting a SRO gain peak in the significant region 3q26.2 only. BAC clone segments are matched to their size. All genes in the region are displayed and those in the significant region are highlighted in yellow. C) Frequency plot of chromosome X. The significant regions are highlighted in grey, and the gene *SH3KBP1 *that was explored with QPCR is shown. D) Frequency plot of chromosome 9. The significant regions are highlighted in grey, and the gene *SH3GL2 *that was explored with QPCR is shown. In all frequency plots: resistant cases (black line) versus sensitive cases (red line).

For the two response groups, the average frequency of altered genome was 38% in resistant cases, whereas 26% in sensitive cases (Figure 1). The difference was statistically significant (*P *= 0.044). Exploring the genetic alteration pattern in the ovarian tumors in relation to chemotherapy response revealed regions in four chromosomal arms that differed significantly between the sensitive and resistant cases, and that were more frequent in the resistant tumors (Table 2). Gain in region 3q26.2 was significantly more frequently detected in the resistant cases. It is a small region (150 kbp) containing only two known genes (*EVI1 *and *MDS1*). The region was the smallest region of overlap (SRO) in 3q26 and 65% of the resistant cases displayed gain in the region; whereas 20% of the sensitive cases. Some tumors exhibited gain peaks exclusively in the SRO (Figure 2B) and some exhibited peaks in the close proximity of the significant region. Further, losses in three regions in chromosome arm Xp (Xp22.2-22.12, Xp22.11-11.3, Xp11.23-11.1) were significantly more frequent in resistant cases than sensitive cases (Table 2). The regions are rather large and most tumors displayed alterations extending along a major part of the p-arm, and along the q-arm as well (Figure 2C). The alteration pattern was simplex and none displayed homozygous deletions. The significant regions together contain 265 known genes with varying types of functions. Additionally, losses in three regions in chromosome arm 9p (9p22.3, 9p22.2-22.1, 9p22.1-21.3) were significantly associated with resistance (Table 2). The three closely situated regions are quite small and contain 11 known genes. Alterations in the regions were rather large and many extended along half the p-arm (Figure 2D). A further significant region associated with resistance was 6q11.2-12 (Table 2). It is a 4.85 Mbp large region harboring 16 known genes, of which none seems to be of obvious interest.

**Table 2:** Significant regions

cytoBand (+ gain, - loss)	Mbp startPos (BAC)	Mbp endPos (BAC)	Size (Mbp)	cases resistant (%)	cases sensitive (%)	*P*-value	No. of genes
+ 3q26.2	170150001 (RP11-152C17)	170300002 (RP11-252J1)	0.15	65	20	0.0095	2

- 6q11.2-12	63400001 (RP11-692G18)	68250002 (RP11-164N24)	4.85	40	0	0.0033	16

- 9p22.3	15100001 (RP11-271D19)	16150002 (RP11-141K7)	1.05	45	5	0.0084	6
- 9p22.2-22.1	17750001 (RP11-601F21)	18550002 (RP11-269F13)	0.8	45	5	0.0084	2
- 9p22.1-21.3	19650001 (RP11-61D22)	20800002 (RP11-66P3)	1.15	45	5	0.0084	3

- Xp22.2-22.12	15250001 (RP11-438H12)	20750002 (RP11-451E9)	5.5	60-65	15	0.0031-0.0079	39
- Xp22.11-11.3	24050001 (RP11-79B3)	46900002 (RP11-571E6)	22.85	60-70	15-20	0.00077-0.0095	141
- Xp11.23-11.1	49250001 (RP11-122N23)	58250002 (RP11-96A5)	9	60-65	10-15	0.00077-0.0079	85

Regions exhibiting statistical significance (*P *< 0.01) between sensitive and resistant cases. The intervals in frequency numbers and *P*-values are due to CNA gaps inside the significant regions.

**Figure 3:**
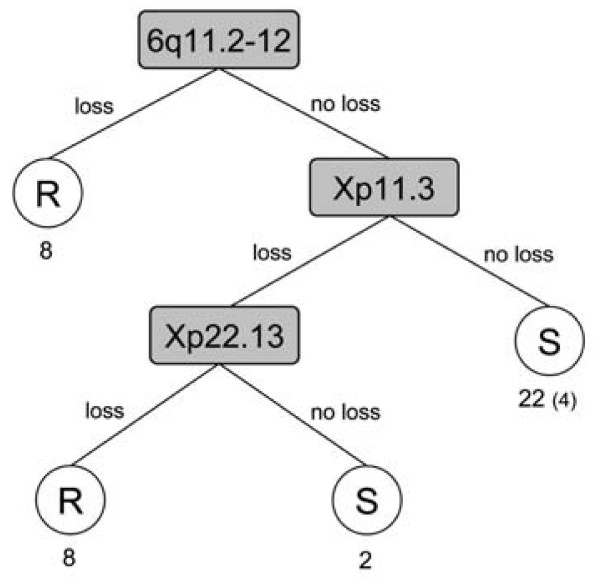
**Decision tree**. A decision tree based on the significant regions chose 6q11.2-12, Xp11.3 and Xp22.13 as the best combination of classifiers. Numbers beneath the circles are the number of cases classified in each group. Numbers in brackets are incorrectly classified cases if any. R = resistant, S = sensitive.

**Figure 4:**
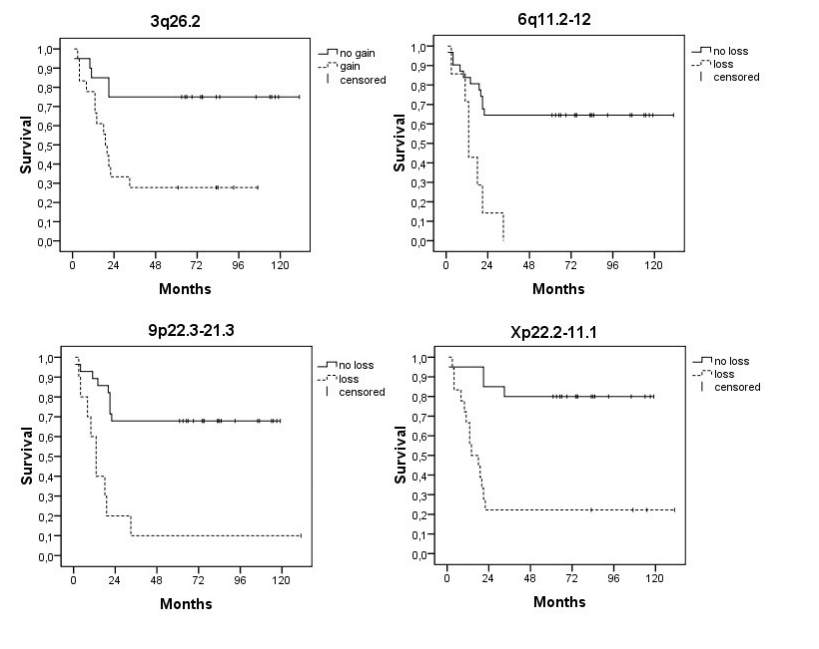
**Survival curves**. Survival curves for the significant regions in 3q26.2, 6q11.2-12, 9p22.3-21.3, and Xp22.2-11.1. The lines represent the survival of patients whose tumors exhibit or do not exhibit the significant alterations in the respective regions.

A decision tree was generated based on the significant regions, and regions 6q11.2-12, Xp11.3 and Xp22.13 were picked out as the best combination of classifiers (Figure 3). It classified all sensitive cases and 16/20 resistant cases correct with a total correct classification of 90%, and in the leave-one-out cross-validation it showed an accuracy of 78%. When testing the decision tree on a published data set from Partheen and colleagues [27], samples with alterations in the decision tree regions were classified correctly at a high frequency (88% non-survivors correctly for the first node 6q11.2-12, and 82% for the third node Xp22.13) whereas samples without any of the alterations were poorer classified (37% correctly classified). To illustrate the value of the significant regions for patient survival, Kaplan-Meier curves are shown in figure 4.

The much studied and commonly altered region 7q21.12, harboring the multidrug resistance gene ABCB1, was scrutinized in the present material though it did not exhibit significance. Alterations in the region were scarce, and those present did not vary between sensitive and resistant cases; no amplifications or homozygous deletions were found in the region

### QPCR

In the significant genomic regions established by array CGH we located four genes of specific interest for ovarian cancer and chemotherapy response; *EVI1*, *MDS1*, *SH3KBP1 *and *SH3GL2*. The genes *MDS1 *and *EVI1 *at 3q26.2 have been shown by others to be of great interest for ovarian cancer, and have also been implicated in paclitaxel resistance [30-33]. The gene *SH3KBP1*/*CIN85*, located at Xp22.1-21.3, is an essential part of the complex controlling endocytosis of the epidermal growth factor receptor (EGFR) [34], which has been implicated in paclitaxel and cisplatin response [35,36]. Additionally, the protein of the gene *SH3GL2*/*Endophilin A1*, located in 9p22.2-22.1, binds to SH3KBP1 in the complex that control endocytosis of EGFR [34]. Therefore, these genes were selected for gene expression analysis.

**Figure 5:**
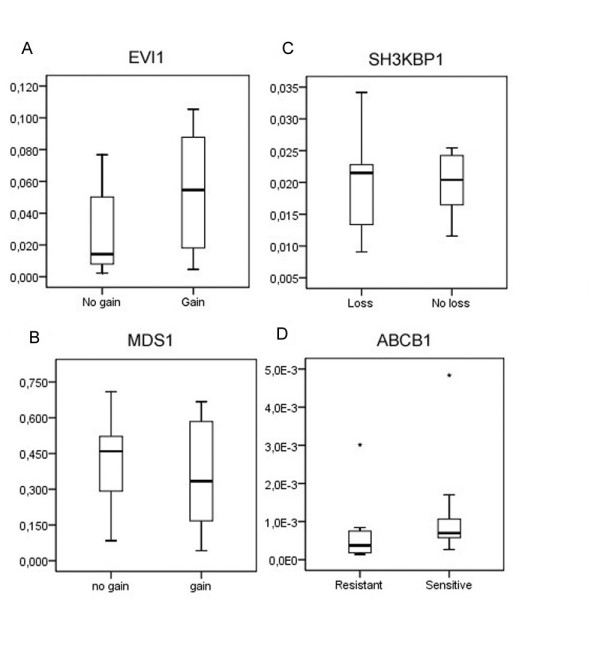
**Expression data**. Relative mRNA expression for genes analyzed with QPCR. Each plot shows the median (centre lines), interquartile ranges (boxes), largest and smallest values (whiskers) that are not outliers (circles), or extreme values (stars) within a category. Samples for the genes *EVI1 *(A), *MDS1 *(B), and *SH3KBP1 *(C) are grouped according to the corresponding CNA exhibiting significance with array CGH. Samples for the gene *ABCB1 *(D) are grouped according to chemotherapy response.

**Figure 6:**
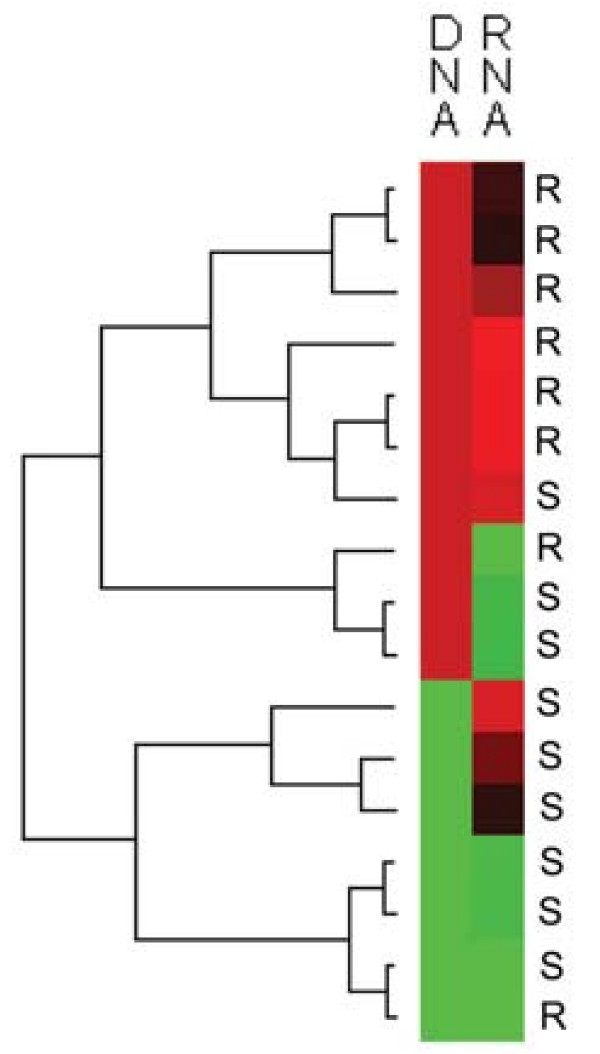
**DNA-RNA correlation for *EVI1***. A heatmap illustrating the mRNA and DNA correlation for the *EVI1 *gene and its locus 3q26.2. Red represents gain and high mRNA expression, respectively, whereas green represents no gain and low expression. Black represents intermediate levels of mRNA expression. In the left panel, tumors were clustered hierarchically according to similarity, using both copy number and expression level in the distance calculation (Euclidean). The chemotherapy response status of the samples is indicated vertically for each sample respectively. R = resistant case, S = sensitive case.

Samples were separated according to the corresponding CNA pattern for each gene, and we aimed to explore and compare the relative gene expression between these groups. Unfortunately, high quality RNA was achievable from only 17 of the 40 tumor samples, thus weakening the results obtainable. The relationships between gene expression and CNA are illustrated in figure 5. Concerning *EVI1*, the average relative expression differed between the samples with gain and without gain, with higher expression in the gain group (1.8 times higher). The difference was borderline significant (*P *= 0.068). Figure 6 illustrates the correlation between DNA and mRNA for the *EVI1 *gene. Expression of the *MDS1 *gene was detected in all samples; however there was no significant difference between samples with gain and without gain (Figure 5B). Nor did we find a significant correlation to the array CGH findings for the *SH3KBP1 *gene (Figure 5C). Further, the gene expression of *SH3GL2 *was generally very low, undetectable even in some samples. Therefore, accurate and reliable calculations were impaired. A slight tendency was noticed however; the group with DNA losses exhibited lower relative mRNA expression than the group without losses. Moreover, the expression levels of the four genes investigated did not differ significantly when grouped according to resistance.

In addition, we explored gene expression of the much studied multidrug resistance gene *ABCB1 *(also known as P-glycoprotein/P-gp or multidrug resistance 1/MDR1), and compared the relative expression between sensitive and resistant groups (Figure 5D). The overall expression of *ABCB1 *was quite low, and there was no significant difference between sensitive and resistant cases. If scrutinizing the data, one might see a tendency that the resistant group exhibit lower expression on average than the sensitive group.

When studying the pattern of CNA co-occurrence for the *SH3KBP1 *and *SH3GL2 *loci, losses in any of the two loci occured in 14/20 (70%) of the resistant cases, with 8 cases of co-losses, and 6 cases with loss in only one of the two loci. The sensitive cases exhibited only 1 co-loss and 2 cases with one of the two loci lost.

## Discussion

The majority of ovarian cancer patients is diagnosed with advanced stage disease of serous histology and treated with combination chemotherapy paclitaxel/carboplatin. In the present investigation we selected only stage III serous primary specimens from patients homogenously treated with adjuvant paclitaxel/carboplatin in order to refine the analysis. Using array CGH, we detected specific genetic alterations associated with resistance, of which some might be potential predictive markers of chemotherapy resistance in advanced ovarian cancer.

Several non-overlapping single genes, gene profiles and CNAs have been suggested as potential markers for chemotherapy response in ovarian cancer [12,13,37-39]. To our knowledge, there are only a few reports on chemotherapy resistance in ovarian cancer using array CGH [12-15]. There are also prior studies using conventional metaphase CGH to investigate platinum resistance in ovarian cancer [16-19]. Unfortunately, results are diverse. The disagreement, however, can partly be explained by the use of various CGH platforms, variations in resistance classifications, heterogeneous tumor materials, and the use of various cell lines. It also emphasizes the difficulty of investigating chemotherapy resistance in ovarian cancer. Hence, additional studies are needed in order to clarify the complex pattern of genetic alterations associated with chemotherapy response in ovarian carcinomas and to identify reliable predictive markers.

In the current study, we associated CNAs in four chromosomal arms with chemotherapy resistance in stage III ovarian serous carcinomas. Gain in the small region at 3q26.2 was significantly established as frequently more present in resistant cases (65%) than sensitive cases (20%). 3q26 is recurrently found amplified in various cancer types including ovarian carcinoma [40-42]. In concordance with our results, Nanjundan and colleagues identified the ~2 Mbp wide region at 3q26.2 containing *EVI1 *and *MDS1 *to be the most frequent region of copy number gain in an ovarian tumor material [30]. Additionally, Kim and colleagues found gain in 3q26.2 in more than 70% of serous ovarian cancer specimens; equally abundant in sensitive and resistant cases [13]. The two genes in the significant region are of great interest. *EVI1 *is an oncogene frequently associated with leukemogenesis [43], and also with other cancer types such as lung- and endometrial cancer [44,45]. It has been found over expressed in ovarian cancer and implicated in ovarian carcinogenesis [30-32]. Interestingly, Liu and colleagues showed that the protein Evi1 inhibited paclitaxel-mediated apoptosis, thus causing resistance [33]. In this context, our finding of gain in 3q26.2 as overrepresented among resistant cases is highly interesting. Further, Sunde and colleagues found a significant correlation between gene copy number and *EVI1 *gene expression, and suggested that enhanced expression of *EVI1 *can partly be explained by increased gene copy number [31]. We did not detect such a strong correlation, but a tendency was noticed in that direction, and enlarged studies scrutinizing *EVI1 *gene and protein expression may elucidate its role in ovarian cancer and chemotherapy response. Additionally, among the tumors exhibiting gains in 3q26, some display a gain peak specifically in the significant region 3q26.2, whereas others display gains extending over a larger region, and a few exhibit peaks in the surrounding regions. This complex pattern of CNAs suggests that more than one driver might exist for the 3q26 gain, as correspondingly detected and stated by others [30]. *MDS1 *was first identified as a component of the AML1-MDS1-EVI1 fusion transcript in myeloid leukemia [46], and very little is known about the gene product when not in a fusion transcript. MDS1 was expressed in the current ovarian tumor material, but the expression results did not correlate with the findings on the DNA level, nor resistance.

Losses in three regions in chromosome arm Xp (Xp22.2-22.12, Xp22.11-11.3, Xp11.23-11.1) were associated with chemotherapy resistance in the current study. The alterations detected here were large, stretching along the whole chromosome in some cases. The X chromosome has not been explored to the same extent as the rest of the genome by CGH due to the use of male reference in several studies. Losses in the X chromosome has been found in cisplatin-resistant cell lines [14-17]. When scrutinizing the 265 known genes in the significant regions found in Xp, our attention was drawn to *SH3KBP1*, also known as *CIN85*. Loss of the *SH3KBP1 *locus as found in our investigation could be a form of resistance mechanism by inhibiting EGFR endocytosis and thus increasing EGFR signaling, which might reduce sensitivity to paclitaxel and/or platinum drugs [35,36]. When exploring mRNA expression levels of *SH3KBP1 *we did not find a difference between samples with loss and no loss, nor resistance. However, we were only able to investigate a small subset of the tumor material, and further studies of the gene should be encouraged on both mRNA and protein expression levels.

Additionally, three small regions of loss in chromosome arm 9p (9p22.3, 9p22.2-22.1, 9p22.1-21.3) were associated with resistance. In ovarian cancer, allelic imbalances are commonly detected in 9p [41], but the region have to our knowledge not been associated with chemotherapy resistance earlier. The gene *SH3GL2 *located in 9p22.2-22.1, also known as *Endophilin A1*, is as *SH3KBP1 *also part of the complex controlling endocytosis of EGFR [34]. Thus, equally interesting in association to chemotherapy resistance as *SH3KBP1*. Unfortunately, the gene showed generally low expression in the ovarian tumor samples and reliable calculations were unfeasible. Still, continued exploration of the gene and its locus should be of interest due to its strong associations to EGFR and chemotherapy response.

We find it very interesting that the proteins of the genes *SH3KBP1 *and *SH3GL2*, located in two different regions exhibiting significance in the current study, both have been shown to physically bind and interact in the complex controlling endocytosis of EGFR [34]. Losses in any of the two loci were found in 70% of the resistant tumors.

Overall, mRNA expression indicated only weak DNA copy number dependence in our study. However, we were only able to obtain high quality RNA from a subset of the tumor material (17/40) which obviously might influence the results. Additionally, further exploration of genes in the significant regions would be of interest. Reliable genetic alteration profiles, however, can be of great interest as predictive markers of patient outcome regardless of its influence on gene expression; and DNA is relatively easy to handle and more stable than RNA.

The decision tree that was generated based on the significant regions, specifically discerned the regions 6q11.2-12, Xp11.3 and Xp22.13 as a good combination of classifiers and predictors of chemoresistant or chemosensitive disease in our tumor material (Figure 3). Losses in the significant region 6q11.2-12 were found exclusively in the resistant tumors, and as shown by the decision tree loss in 6q11.2-12 classified 8 resistant tumors at once. Additional losses in Xp11.3 and Xp22.13 further classify 8 resistant cases. Conversely, lack of these changes classified all sensitive cases correct. This indicates a specific importance of these genomic alterations, and region 6q11.2-12 is therefore of great interest as a predictive marker of chemoresistant disease. As mentioned above, the alterations detected in Xp were not restricted to the significant regions in the majority of cases. Thus, losses in general in Xp ought to be considered interesting for chemotherapy resistance. We further tested the decision tree on another published ovarian tumor material with corresponding stage and histology but with a different combination treatment (carboplatin, farmorubicine and cyclophosphamide) and survival as end point [27]. When scrutinizing the tree, samples exhibiting alterations in the decision tree regions were correctly classified at a rather high frequency (88% and 82%, respectively), whereas samples lacking alterations in the regions were poorly classified (37%). This is in concordance to our material, where the only misclassifications were samples without alterations in the regions. It suggests that alterations in these regions are of importance for the outcome in advanced stage ovarian serous tumors. Tumors without alterations in these regions, on the other hand, need further characterization. Additionally, all resistant cases in our study died of their disease and all sensitive cases exhibited more than five-year survival; and the importance of the significant regions in relation to patient survival is shown by the survival curves in figure 4. Since the decision tree contains regions in chromosome X, and many studies do not analyze X as mentioned above, testing of the tree in further published materials is unfortunately hampered.

We detected a significantly higher frequency of genomic alterations in resistant specimens than in sensitive specimens, which has been reported in similar studies [12-14]. This elevated frequency of genetic alterations might present an advantage for the resistant tumors, and help them to adapt in the hostile chemotherapy environment.

The phenomenon of multidrug resistance (MDR) has been shown to effect paclitaxel [5]. However, the relevance of MDR in ovarian cancer treatment is not clear [47]. A number of reports have focused on multidrug resistance in ovarian carcinoma, and specifically on the drug efflux pump P-glycoprotein, whose gene *ABCB1 *is situated in 7q21.12 [48-50]. The gene has been found over expressed in paclitaxel-resistant ovarian and lung cancer cell lines [48,49]. In the current study, we took the opportunity to study *ABCB1 *in our primary ovarian tumor material. *ABCB1 *showed generally low expression and CNAs in 7q21.12 were infrequent; no differences were seen between sensitive and resistant cases, which is in concurrence with others [51].

## Conclusion

In conclusion, we identified gains in 3q26.2, and losses in 6q11.2-12, 9p22.3-21.3, and Xp22.2-11.1 to be associated with chemotherapy resistance, suggesting that these CNAs might have a potential as predictive markers of chemoresistant disease in patients with advanced ovarian serous cancer. Identifying a high-risk group of patients that will exhibit poor response to conventional chemotherapy could lead to a different treatment regiment already at first-line therapy and a special follow-up of these patients. However, this is a pilot study with a small material and further studies are needed to evaluate and verify the results. Still, our findings contribute to an increased understanding of the genetic alterations in chemoresistant ovarian serous carcinoma.

## Abbreviations

CGH: comparative genomic hybridization; QPCR: quantitative real-time polymerase chain reaction; CNA: copy number alteration; WHO: world health organization; FIGO: international federation of gynecology and obstetrics; BAC: bacterial artificial chromosome; BASE: bio array software environment; Mbp: megabasepair; kbp: kilobasepair; MDR: multidrug resistance; EVI1: ectopic viral integration site-1; MDS1: myelodysplastic syndrome 1; SH3GL2: SH3-domain GRB2-like 2; SH3KBP1: SH3-domain kinase binding protein 1; ABCB1: ATP-binding cassette, sub-family B, member 1

## Competing interests

The authors declare that they have no competing interests.

## Authors' contributions

LÖ participated in the design of the study, performed and analysed the array CGH analysis, analysed the QPCR data, and drafted the manuscript. KL and KP have been involved in interpretation of the data, drafting the manuscript and revised it critically. UD has been involved in and performed the array CGH analysis and RNA extraction. BO has performed the statistics and revised the manuscript critically. KS has been involved in interpretation of the data, provided clinical information and revised the manuscript critically. GH initiated the project, provided clinical information, and was involved in drafting the manuscript. All authors read and approved the final manuscript.

## Pre-publication history

The pre-publication history for this paper can be accessed here:

http://www.biomedcentral.com/1471-2407/9/368/prepub

## Supplementary Material

Additional file 1 **Clinical characteristics**. Clinical characteristics of the forty patients. Tumors were analyzed routinely by flow cytometry for DNA content. W = well differentiated; M = moderately differentiated; P = poorly differentiated; † = deceased; S = sensitive; R = resistant; An = aneuploid; Di = diploid Click here for file

Additional file 2 **Primer sequences**. Primers sequences for the target genes in the QPCR analysis. Click here for file

Additional file 3 **Array data**. Segmented ternary array CGH data. All clones were designated lost, not changed, or gained, giving a ternary scale (-1, 0, 1). The segmented BAC clone data was transformed into a virtual probe set with probes spaced at every 50 kbp throughout the entire genome by associating each probe to the closest virtual probe. Click here for file
